# Desensitization With Imlifidase: Overcoming Immunological Barriers in Kidney Transplantation

**DOI:** 10.1016/j.xkme.2025.101076

**Published:** 2025-08-05

**Authors:** Paul José Hernández-Velasco, Eduardo Gutiérrez Martínez, Natalia Polanco Fernández, Maria Esther González Monte, Celia González-García, Esther Mancebo Sierra, Amado Andrés Belmonte

**Affiliations:** 1 Department of Nephrology, Hospital Universitario 12 de Octubre, Madrid, Spain; 2 Department of Nephrology, Hospital Universitario 12 de Octubre, Instituto de Investigación Hospital 12 de Octubre (imas12), Madrid, Spain; 3 Department of Immunology, Hospital Universitario 12 de Octubre, Madrid, Spain; 4 Department of Nephrology, Hospital Universitario 12 de Octubre. Instituto de Investigación Hospital 12 de Octubre (imas12). Department of Medicine, Universidad Complutense, Madrid, Spain

**Keywords:** Desensitization, donor-specific antibodies, highly sensitized patients, imlifidase, kidney transplant

## Abstract

Highly sensitized patients without compatible living donors face prolonged waiting times for a transplant. Prioritization and desensitization strategies often prove insufficient because of logistical challenges, extended treatments, and increased infection risk. The new drug imlifidase offers an opportunity for these patients by rapidly removing IgG antibodies, enabling desensitization when a donor is available. However, real-word experience remains limited. Here, we present the first case in Spain, outside of a clinical trial, in which imlifidase desensitization allowing a kidney transplant in a woman with a calculated panel reactive antibody >99.99%, requiring a second kidney transplant after 13 years on dialysis. After anti-human leukocyte antigen antibodies delisting (<20,000 mean fluorescence intensity [MFI], responders to dilution and noncomplement fixing), she received a deceased-donor kidney against whom she had 6 donor-specific antibodies (DSAs; ranging from 3,049 to 12,001 MFI; targeting human leukocyte antigen class I and II) and a positive flow cytometry crossmatch—all becoming negative after treatment with imlifidase. Early post-transplant DSA rebound was managed with conventional desensitization and anti-C5 blockade. Short-term outcomes were encouraging, with stable kidney function and significant DSA reduction at 9 months. This case highlights the potential of imlifidase in highly sensitized patients; however, long-term studies remain essential to optimize monitoring and concomitant desensitization protocols.

Highly sensitized patients, especially those with a calculated panel reactive antibody (cPRA) greater than 98%, often face prolonged waiting times for a transplant, despite prioritization programs, low-risk antibody delisting, and desensitization strategies. Facilitating transplant access for some patients involves accepting transplantation in the presence of DSAs, even when cell-based crossmatch tests are positive, with the consequent increase in immunological risk and a worse prognosis.^1^ Therefore, a positive crossmatch result is generally considered a contraindication for kidney transplantation unless successful desensitization is achieved.

Imlifidase, a recently available desensitization therapy, rapidly degrades immunoglobulin G (IgG), including anti-human leukocyte antigen (anti-HLA) donor-specific antibodies (DSAs), potentially enabling transplantation in high-risk cases. Here, we present the first case in Spain, outside of a clinical trial, in which desensitization with imlifidase allowed a kidney transplant in a highly sensitized patient with a positive flow cytometry crossmatch (FC-XM) and multiple high-level DSAs.

## Case Report

A 51-year-old White woman with a history of chronic hepatitis B infection and chronic kidney disease secondary to chronic glomerulonephritis. She started hemodialysis in 1997 and received a kidney transplant from a related living donor in 2002. However, because of chronic allograft injury, she resumed hemodialysis in 2011.

The patient was highly sensitized, with a cPRA >99.99%. In 2015, she was included in the Spanish exchange program for highly sensitized patients (PATHI),^2^ without finding a compatible donor due to the presence of DSA against all registered donors. In 2020, antibodies with a mean fluorescence intensity (MFI) <5,000 were delisted, but this was insufficient to allow transplantation. In January 2024, an additional delisting targeted antibodies with a high MFI (<20,000) that responded to dilution studies and were noncomplement fixing (single antigen beads-C1q negative). This approach lowered her “prohibited antigens” cPRA to 98%, increasing the likelihood of suitable donor offers.

Preventive desensitization with imlifidase was considered to mitigate the deleterious effects of “possible” high-risk DSAs and the possibility of a positive cell-based crossmatch. After delisting, the patient received 2 organ offers, both declined due to unsuitable donor characteristics. In June 2024, 154 days after delisting, a third offer was accepted. The donor was a 24-year-old male who died of circulatory arrest and met no expanded criteria.

The recipient had 4 HLA mismatches with the donor and 6 DSAs against DP1 (12001 MFI), DR53 (10060 MFI), DR7 (9326 MFI), DQ2 (8194 MFI), B65 (7302 MFI), and B44 (3049 MFI). FC-XM and complement-dependent cytotoxicity (CDC-XM), performed on T and B lymphocytes from the spleen, were positive. However, the positive CDC-XM result was reversed with dithiothreitol, indicating an IgM-mediated reaction.

**Figure 1:**
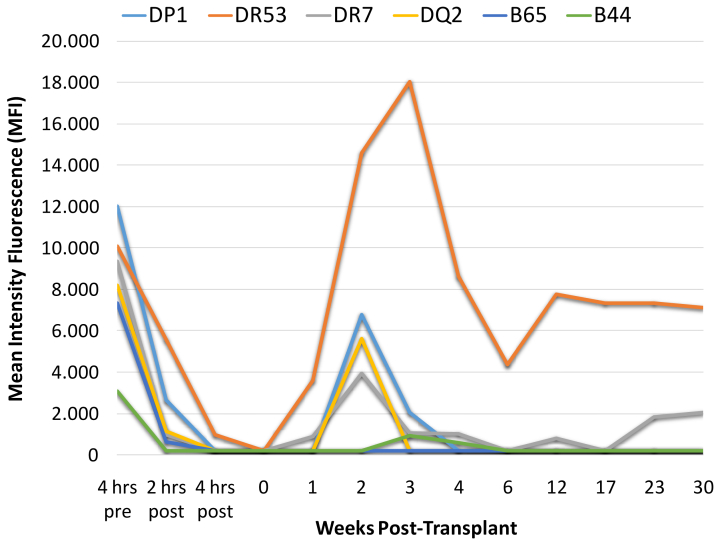
Evolution of DSAs MFI before and after transplantation.

A single dose of imlifidase (0.25 mg/kg) was administered. After 2 hours, FC-XM turned negative, and CDC-XM remained positive. This was reversed by dithiothreitol, enabling transplantation. Postdose DSAs levels decreased significantly, with only DR53 remaining detectable (MFI 953) (Fig 1).

**Figure 2:**
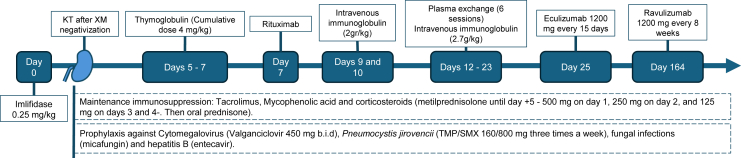
Immunosuppressant treatment regimen over time. A single dose of imlifidase (0.25 mg/kg) was administered. After 2 hours, FC-XM turned negative. Induction therapy with anti-thymocyte globulin (ATG) was delayed. Instead, the patient received high-dose methylprednisolone (500 mg on day 1, 250 mg on day 2, and 125 mg on days 3 and 4). ATG was initiated on day 5 (total dose 4 mg/kg). Followed by rituximab on day 7 (1 g) and intravenous immunoglobulin (IVIg) on days 9 and 10 (total dose 2 g/kg). Maintenance immunosuppression consisted in tacrolimus, mycophenolate mofetil, and oral prednisone. Standard prophylaxis against cytomegalovirus and *Pneumocystis jirovecii* was administered, along with ceftriaxone, micafungin and entecavir for the patient's history of chronic hepatitis B infection. After DSA rebound was treated with 6 sessions of plasma exchange and IVIg (cumulative dose of 2.7 g/kg) and C5-inhibiting therapy.

Due to imlifidase's degradation of IgG, induction therapy with anti-thymocyte globulin was delayed and initiated on day 5. Instead, the patient received high-dose methylprednisolone between day 1 and 4. Induction therapy also included rituximab on day 7 and intravenous immunoglobulin (IVIg) on days 9 and 10. Details of immunosuppressive and prophylactic treatment are included in Figure 2.

The cold ischemia time was 22 hours. Post-transplant kidney function was immediate, with serum creatinine levels stabilized between 0.9 and 1.2 mg/dL. On day 10, the patient experienced a significant rebound of DSA against HLA DR53 (14603 MFI), DP1 (6750 MFI), DQ2 (5613 MFI), and DR7 (3909 MFI), coinciding with reduced urine output (< 1000 mL) and worsening serum creatinine levels (1.4 mg/dL). A kidney biopsy was planned to assess microvascular inflammation, but the patient declined the procedure. Empirical treatment with 6 sessions of plasma exchange and IVIg (cumulative dose of 2.7 g/kg) led to a marked reduction in DSAs, with only anti-DR53 remaining detectable. Given the precedent of historical complement-binding DSAs, complement C5-inhibiting therapy with eculizumab was instituted on day 25. Because a long-term complement inhibition was planned, she was switched to ravulizumab on day 164 (Fig 2).

At 9 months of follow-up, kidney function remained stable, with serum creatinine levels ranging from 0.8 to 1.2 mg/dL. The last available analysis showed serum creatinine levels of 1.02 mg/dL and a glomerular filtration rate of 63 mL/min per 1.73 m^2^. The single antigen beads assay shows persistence of DSA against DR53 (7120 MFI) and DR7 (2015 MFI), without evidence of other DSA (Fig 1).

## Discussion

Prioritization programs like PATHI have improved access to kidney transplantation for highly sensitized patients. Nevertheless, patients with a cPRA of 100% have significantly lower transplant rates than those with a cPRA of 98% to 99%.^2^ In such cases, delisting strategies aim to minimize immunological risk and improve transplant accessibility,^3^ but patients with particularly deleterious antibodies, require preventive desensitization. Traditional desensitization methods, based on plasma exchange, IVIg, and rituximab, have shown effectiveness in living-donor kidney transplantation^3^^,^^4^ but are less feasible for deceased donors because of unpredictability donor availability, the need for prolonged treatment cycles, and an increased infection risk.^5^ Traditional desensitization may benefit patients with low-MFI anti-HLA antibody profiles who respond well to dilution studies,^6^ but it is far less effective for patients with high MFI profile, such as the case presented here.

Imlifidase addresses these challenges. This cysteine protease, produced by *Streptococcus pyogenes,* cleaves human IgG, separating the F(ab')_2_ fragment from the Fc fragment, degrading both vascular and extravascular IgG as well IgG-type B-cell receptors,^7^ leading to crossmatch negativization and enabling transplantation with an available donor. In Spain, imlifidase was approved in 2023 for desensitizing highly sensitized adult patients undergoing kidney transplantation with a positive crossmatch (CDC-XM or FC-XM) against a deceased donor. Eligibility criteria include a cPRA ≥ 99% or ≥ 98% with 2 or 4 years in PATHI or regional prioritization program, or a patient who cannot continue dialysis.^8^

The main advantage of imlifidase over traditional desensitization is its rapid action and effectiveness. It cleaves plasma IgG into single-cleaved IgG within minutes and fully separates the F(ab')_2_ and Fc fragments within 6 hours^9^, overcoming logistical barriers in deceased-donor kidney transplantation. This is exemplified in the present patient, in which the crossmatch was reversed to negative within 2 hours, allowing transplantation.

Clinical trials have evaluated imlifidase in 46 highly sensitized kidney transplant patients,^10^, ^11^, ^12^ 39 of whom had a positive cell-based crossmatch (CDC-XM and/or FC-XM) and a median DSA of 7,791 MFI (IQR: 4,108-16,320).^13^ DSAs rebound can occurs within 3-14 days after infusion, with interindividual variability^10^^,^^13^ and acute antibody-mediated rejection (ABMR) occurred in 39% of cases, mostly within the first 6 months.^12^ The 5-year global survival rate was 90%, and allograft survival was 82%.^14^

Reports of imlifidase use outside clinical trials are limited. Kamar et al^15^ described 9 patients with a cPRA ≥ 99% and positive FC-XM; 2 also had positive CDC-XM. Within 3 months, 5 patients experienced DSA rebound, including the 2 patients with a positive CDC-XM. Notably, only those with rebound developed clinical ABMR (2 clinical, 2 subclinical), but no graft loss was reported. Only one patient had a sum DSA MFI >30,000 and, like our patient (49,032 MFI), experienced early rebound. Their patient developed clinical ABMR, whereas our patient could not be confirmed because of lack of biopsy. Despite no histology, the high pretreatment DSA load, early rebound, and worsening kidney function guided post-transplant desensitization. This suboptimal scenario highlights the need for strong patient commitment, including prior consent to protocol and indication-based biopsies.

In other European countries such as France, imlifidase is authorized for use in patients with a positive virtual crossmatch and immunodominant DSA >6,000 MFI.^16^ In Spain, a positive cell-based crossmatch is required, making access more restrictive and limited to patients with greater immunological risk.^8^^,^^17^ It is not unusual to obtain negative cell-based crossmatches even in the presence of high-MFI DSA, and in these cases, imlifidase treatment is not allowed in Spain.

Our case shows a novel desensitization strategy for highly sensitized patients with multiple high-MFI anti-HLA antibodies which requires a high-risk delisting that probably will lead to a positive cell-based crossmatch. Although the effectiveness and rapid action of imlifidase are unquestionable, DSA rebound is frequent and clinically associated with ABMR, particularly early post-transplant, highlighting the need for additional desensitization strategies to manage it.

Finally, CDC-XM testing with dithiothreitol remains crucial to differentiate IgM-mediated reactions. IgM-DSA can fix complement and result in positive CDC-XM results, but dithiothreitol degrades IgM, allowing accurate IgG evaluation. Because imlifidase exclusively degrades IgG, proper CDC-XM interpretation is essential post-treatment.

This study has limitations. First, the lack of a biopsy, declined by the patient, makes ABMR assessment challenging. Additionally, the short follow-up period limits the ability to assess long-term efficacy and safety. Case reports are inherently subject to selection bias, and individual outcomes may not reflect the full spectrum of clinical responses; thus, broader conclusions should be drawn with caution. Given its high cost and limited availability, imlifidase use should be carefully individualized, with a clear plan for managing potential DSA rebound through timely concomitant treatment. Further studies are needed to determine long-term outcomes and refine immunological monitoring and desensitization protocols for these patients.
